# Supplementary material for: A randomised, controlled, observer-masked trial of corneal cross-linking for progressive keratoconus in children: the KERALINK protocol
Source: BMJ Open. 2019 Sep 12;9(9):e028761. doi: 10.1136/bmjopen-2018-028761 (PMC6747657; doi:10.1136/bmjopen-2018-028761)
Supplement: Author's manuscript [file bmjopen-2018-028761.draft_revisions.pdf]

BMJ Open is committed to open peer review. As part of this commitment we make the peer review history of every article we publish publicly available.

When an article is published we post the peer reviewers' comments and the authors' responses online. We also post the versions of the paper that were used during peer review. These are the versions that the peer review comments apply to.

The versions of the paper that follow are the versions that were submitted during the peer review process. They are not the versions of record or the final published versions. They should not be cited or distributed as the published version of this manuscript.

BMJ Open is an open access journal and the full, final, typeset and author-corrected version of record of the manuscript is available on our site with no access controls, subscription charges or pay-per-view fees (http://bmjopen.bmj.com).

If you have any questions on BMJ Open's open peer review process please email info.bmjopen@bmj.com

BMJ Open

A randomised, controlled, observer-masked trial of corneal cross-linking for progressive keratoconus in children: the KERALINK trial design and methodology

Journal:	*BMJ Open*
Manuscript ID	bmjopen-2018-028761
Article Type:	Protocol
Date Submitted by the Author:	21-Dec-2018

Complete List of Authors:	Chowdhury, Kashfia; University College London, Comprehensive Clinical Trials Unit<br>Dore, Caroline; University College London, Comprehensive Clinical Trials Unit<br>Burr, Jennifer; University of St Andrews<br>Bunce, Catey; kings College london, Primary Care and Public Health Sciences<br>Raynor, Mathew; Royal Hallamshire Hospital, Ophthalmology<br>Edwards, Matthew; Royal Hallamshire Hospital, Ophthalmology<br>Larkin, Frank; Moorfields Eye Hospital,

Keywords:	Corneal and external diseases < OPHTHALMOLOGY, Paediatric ophthalmology < OPHTHALMOLOGY, cross linking, keratoconus

SCHOLARONE™
Manuscripts

A randomised, controlled, observer-masked trial of corneal cross-linking for progressive keratoconus in children: the KERALINK trial design and methodology

Chowdhury K[1], Doré C[1], Burr JM[2], Bunce C[3], Raynor M[4], Edwards M[4], Larkin DFP[5] for the Keralink Trial Study Group.

Author affiliations and email addresses

[1] UCL Comprehensive Clinical Trials Unit, London

[2] University of St Andrews, Fife

[3] School of Population Health and Environmental Sciences, King's College London

[4] Royal Hallamshire Hospital, Sheffield

[5] NIHR Moorfields Biomedical Research Centre and NIHR Moorfields Clinical Research Facility, Moorfields Eye Hospital, London, United Kingdom

k.chowdhury@ucl.ac.uk

caroline.dore@ucl.ac.uk

jmb28@st-andrews.ac.uk

catey.bunce@kcl.ac.uk

Mathew.raynor@sth.nhs.uk

Matthew.edwards@sth.nhs.uk

frank.larkin1@nhs.net

Corresponding author

Daniel F P Larkin MD FRCPI FRCOphth

For peer review only

For peer review only - http://bmjopen.bmj.com/site/about/guidelines.xhtml

Moorfields Eye Hospital, City Road, London EC1V 2PD, UK

f.larkin@ucl.ac.uk

Tel 020 7566 2045

Keywords

Cornea, keratoconus, progression, cross linking, topography

Word count 3837 excluding title page and references

For peer review only

ABSTRACT

Introduction The Keralink trial tests the hypothesis that corneal cross-linking treatment (CXL) reduces the progression of keratoconus in comparison to standard care in patients under 17 years old. Keralink is a randomised controlled, observer-masked, multicentre trial in progressive keratoconus comparing epithelium-off CXL with standard care, including spectacles or contact lenses as necessary for best corrected acuity.

Methods and analysis A total of 30 participants will be randomised per group. Eligible participants aged 10-16 years with progressive keratoconus in one or both eyes will be recruited. Following randomisation, participants will be followed up 3-monthly for 18 months. The effect on progression will be determined by K_2 on corneal topography. The primary outcome measure is between-group difference in K_2 at 18 months adjusted for K_2 at baseline examination. Secondary outcomes are the effect of CXL on *(i)* keratoconus progression, *(ii)* time to keratoconus progression, *(iii)* visual acuity, *(iv)* refraction *(v)* apical corneal thickness and *(vi)* adverse events. Patient-reported effects will be explored by questionnaires.

Ethics and dissemination Research Ethics Committee Approval was obtained on 30 June 2016 (ref: 14/LO/1937). Current protocol: v5.0 (08/11/2017). Study findings will be published in peer-reviewed journals.

Trial registration number ISRCTN 17303768.

For peer review only

For peer review only - http://bmjopen.bmj.com/site/about/guidelines.xhtml

STRENGTHS AND LIMITATIONS OF THIS STUDY

- This is the first randomised trial of corneal cross-linking (CXL) in keratoconus in children, in which group disease onset is at an early age, is perceived to be at high risk of progression to corneal transplantation and in which only observational studies have been published.
- A total of 60 patients aged 10-16 years with progressive keratoconus will be randomised to CXL or standard care including spectacles and contact lenses as required for best corrected vision.
- The trial is designed to examine safety and efficacy of CXL in reducing progression, the primary outcome measure being between-group difference in K_2 at 18 months adjusted for K_2 at baseline examination and measured by masked optometrists.
- Secondary outcome measures at 18 months include keratoconus progression, visual acuity, refraction, adverse events and quality of life measurements.
- Follow up to 18 months after randomisation is relatively short and any benefit found following CXL would require longer term analysis of efficacy.

For peer review only - http://bmjopen.bmj.com/site/about/guidelines.xhtml

INTRODUCTION

Keratoconus is characterised by thinning and distortion of the cornea that results in visual loss from complex refractive error and corneal opacification. The prevalence in Europe has been reported as 1:1163[1] and 1:375[2]. The age at initial referral to hospital clinics is the second and third decade (mean age at diagnosis 28 years[2]), with progression until the early 30s in most affected eyes. In its early stages keratoconus causes worsening of vision on account of increasing myopia and irregular astigmatism: spectacle correction provides good visual acuity in early disease only, until increasing irregular astigmatism requires correction with rigid contact lenses for best vision. Patients with more advanced keratoconus lose contact lens-corrected visual acuity on account of corneal opacification and corneal transplant surgery is eventually required in more than 20% of patients[3]. Keratoconus is often more advanced when first diagnosed in children than in adults, with faster subsequent disease progression[4].

The most important parameters used in the assessment of keratoconus are the curvature of the cornea (presented as dioptre power (K)), apical corneal thickness in µm, refraction, and best-corrected visual acuity. Earliest disease can be detected by corneal topography, which demonstrates thinning and irregularity of corneal curvature. Quantification of steepness of the corneal curvature in horizontal, vertical and multiple oblique meridians identifies the meridian of maximum corneal steepness (K_2) and the point of maximum steepness (K_{max}).

While the standard care described above involves treatment of the refractive consequences of keratoconus or replacement of the diseased cornea by a transplant, the concept of stabilising keratoconus and arresting its progression at a stage when there is still good unaided or spectacle-corrected vision is relatively recent. Corneal cross-linking (CXL) increases the stiffness of the cornea, which can arrest the progression of early keratoconus[5]. It is the only current intervention for this purpose. In the epithelium-off CXL procedure corneal epithelium is removed, riboflavin eye drops

For peer review only

administered, and the cornea exposed to ultraviolet (UV) light for 8 or more minutes. CXL has been reported to be effective in arresting keratoconus progression in the majority of treated adult eyes in a number of non-randomised studies (including Henriquez et al. 2011[6], Hersh et al. 2011[7]) and two randomised controlled trials (RCTs) (O'Brart et al 2011[8], Wittig-Silva et al. 2014[9]). In the larger study by Wittig-Silva et al. a significant difference in progression of K_{max} between CXL and control eyes was reported: an improvement in CXL-treated eyes with flattening of K_{max} by -1.03 ± 0.19 D compared to an increase in K_{max} for control eyes of +1.75 ± 0.38 D at 36 months. Adverse effects were not uncommon but mostly transient, including corneal oedema, superficial opacification and recurrent corneal erosions. Despite increasing information in relation to the efficacy of CXL a Cochrane Review conducted in 2015 concluded that evidence for the use of CXL in the management of keratoconus is limited due to the lack of properly conducted RCTs.[10]

In younger subjects three observational studies of CXL in keratoconus patients <19 years have been published, each with limitations but each reporting effectiveness. Caporossi et al. reported an uncontrolled study of 152 keratoconus patients ranging in age from 10 to 18 years, of whom follow-up post-CXL was available on only 61% of patients[11]. Inclusion criteria included several parameters which are well recognised to be characterised by inter-test variability. In this treated patient group, a statistically significant reduction of K_{max} by -0.4 D was found. Vinciguerra et al. reported 40 CXL-treated eyes in patients with progressive keratoconus aged 9-18 (mean 14.2) years in a non-randomised prospective study[12]. Findings included improved visual acuity, reduced myopic spherical equivalent on refraction testing and flattening on keratometry readings compared to pre-CXL. Goodfrooij et al reported progression in 22% within five years of CXL[13]. Although the findings from these studies suggested a beneficial effect of CXL, more robust evidence is required to inform practice. Of note, no randomised trial has been undertaken in young patients. The Keralink trial has been designed to investigate efficacy and safety of the established technique of CXL in progressive keratoconus in the paediatric age group, in which on account of early disease onset there is such

For peer review only - http://bmjopen.bmj.com/site/about/guidelines.xhtml

potential for keratoconus progression. This paper describes the design of the trial, which compares progression of keratoconus in a population of children and young patients randomised to CXL or standard care, and evaluates safety of the intervention in this patient group.

In summary, evidence of effectiveness of CXL is of particular interest in young patients and has specifically been requested by the National Institute for Health and Care Excellence in the United Kingdom. Keralink is a multicentre randomised controlled trial in this patient group evaluating epithelium-off CXL, the technique of CXL which has been demonstrated to be effective in adults. If the trial demonstrates efficacy of CXL compared to standard care, and in particular if CXL is arrests keratoconus progression, this would have important implications for clinical management. Although we intend to follow up the trial patients for several years after the proposed trial concludes in order to ascertain the duration of keratoconus stability, it is clear that arrested progression in a paediatric patient is likely *(a)* to obviate the need for contact lens correction and for later corneal transplant surgery and *(b)* to have correspondingly greater health and cost benefit than if the CXL were undertaken in adults. Trial findings will inform ophthalmologists, optometrists and inform future research and treatment policy.

METHODS AND ANALYSIS

Study design

Keralink is a randomised controlled, observer-masked controlled trial in five centres in the United Kingdom. The study adheres to the tenets of the Declaration of Helsinki and is registered at www.controlled-trials.com (trial registration number: ISRCTN 17303768). It was approved by the UK Health Research Authority, the Medicines and Healthcare Regulatory Agency and ethical approval was granted by the Brent Ethics Committee (reference 16/LO/0913). The trial is supervised by a trial management group, with independent oversight by a trial steering committee and a data monitoring committee. Eligible patients are randomised in a 1:1 ratio to receive either CXL or standard care including spectacles or contact lenses as necessary. Following randomisation, participants are

For peer review only - http://bmjopen.bmj.com/site/about/guidelines.xhtml

followed for 18 months at 3-monthly intervals. Inclusion and exclusion criteria are shown in Table 1. All follow up measurements are performed by masked observers (optometrists) and the treating ophthalmologists are masked as to keratometry values on topography at follow up. Randomisation commenced on October 31 2016 and follow up of the last recruited patient is estimated to complete in mid-2020.

TABLE 1

Keralink inclusion and exclusion criteria

INCLUSION CRITERIA	Age at randomisation: 10-16 years
	Confirmed keratoconus diagnosis
	Progression on Pentacam topography in one or both eyes, steepest corneal meridian (K2) or K_{max} >1.5D
EXCLUSION CRITERIA	Apical scarring
	Cone apex thickness <400µ
	K2 >62.0 D or K_{max} >70.0 D
	Rigid lens wear in both eyes and unable to abstain for 7 days pre-topography examinations
	Down's syndrome

For peer review only

Definition of progression for eligibility

To differentiate true keratoconus progression from measurement artefact or minimal progression, an increase on topography (Pentacam, Oculus GmbH, Wetzlar, Germany) in the steepest keratometry (K_{max}) or in the steepest corneal meridian (K_2) of at least 1.5 dioptres (D) was used as threshold for eligibility in one or both eyes. Based on this, eligibility was defined by an increase from baseline in K_{max} or K_2 of >1.5D between two topography examinations separated by 3 or more months. For each patient the eye with the more advanced keratoconus at baseline will be categorised as the study eye for the primary analysis, unless that eye had undergone prior surgery such as corneal transplantation.

Baseline assessment

At baseline all patients will be assessed as follows.

For peer review only - http://bmjopen.bmj.com/site/about/guidelines.xhtml

On these visits the following assessments will be performed.

- Corneal topography for measurement of corneal power in the steepest meridian (K_2), used for assessment of the primary outcome. To improve repeatability, three measurements of each eye will be taken at baseline and follow-up examinations and the mean used in comparisons.
- Visual acuity (unaided, spectacle- and contact lens-corrected as applicable), logMAR measured using the ETDRS chart at a starting distance of 4m in both eyes
- Refraction, both eyes
- Apical corneal thickness measurement, both eyes
- Quality of life will be assessed by visual function (CVAQC) and generic paediatric health outcome (CHU9D) questionnaires. CVAQC is a 25-item vision specific questionnaire designed for children[14]. CHU9D is a nine-question paediatric generic preference based measure of health outcome which provides a descriptive health profile as well as a utility score and has been validated for self-completion in an adolescent population (11-17 years)[15].

Randomisation and allocation of participants to treatment groups

Randomisation will be by a centralised computer generated randomisation service (*https://www.sealedenvelope.com*). The system is customised to trial requirements, using minimisation with stratification by treatment centre and whether progression is confirmed in one eye or both eyes at randomisation. Following a dedicated consent/screening and randomisation visit for eligible patients and their parents, patients will be randomised to one of two trial arms (Figure 1).

Intervention: CXL

Corneal cross-linking in one or both eyes (according to whether progression is confirmed in one eye or both), under general or local anaesthesia as applicable, followed by standard management. The surgical procedure will be as follows: removal of corneal epithelium with a spatula, administration of riboflavin drops (Vibex Rapid, Avedro Inc., Waltham, Massachusetts, USA) every 2 minutes for 10 minutes, application of pulsed ultraviolet light using standardised parameters of 10mW/cm^2 for a

For peer review only

For peer review only - http://bmjopen.bmj.com/site/about/guidelines.xhtml

5.4J/cm^2 total energy dose administered over 8 minutes. At completion of the procedure one drop of povidone iodine and a therapeutic contact lens will be applied to the treated eye. Management post-CXL is *(i)* proxymetacaine drops every 2 hours and naproxen 250mg twice daily, both as required for analgesia, *(ii)* moxifloxacin 0.5% drops every 6 hours for one week as infection prophylaxis, *(iii)* dexamethasone 0.1% drops every 6 hours for one week, every 12 hours for one week, then fluorometholone 0.1% drops every 12 hours for one week. Patients randomised to CXL will attend for an extra examination at 1 week post-CXL for removal of the contact lens and confirmation of corneal re-epithelialisation.

Comparator: Standard care

The trial control arm is standard management alone, including refraction testing with provision of glasses and/or contact lens fitting for one or both eyes as required for best corrected visual acuity.

Defining keratoconus progression for secondary outcomes

To differentiate true keratoconus progression from measurement artefact, we will define progression as an increase in power in the steepest corneal meridian (K_2) of >1.5 D on corneal topography between two examinations or the requirement for change from spectacle to rigid contact lenses correction of vision, as the latter precludes reliable topography measurements.

Outcome measures

The primary trial outcome measure will be between-group difference in K_2 at 18 months adjusted for K_2 at baseline examination.

Secondary outcomes will be the effect of CXL on

(a) Keratoconus progression (yes/no) defined as >1.5D increase from baseline in K_2, confirmed at subsequent visits *or* keratoconus progression requiring change from spectacle to rigid contact lens correction of vision, which precludes reliable topography measurements

(b) time to keratoconus progression

(c) uncorrected and best corrected visual acuity (logMAR) measured with an ETDRS chart at a starting distance of 4m

For peer review only

For peer review only - http://bmjopen.bmj.com/site/about/guidelines.xhtml

222 *(d)* refraction (measured dioptres spherical equivalent, myopia and astigmatism)

223 *(e)* apical corneal thickness

224 *(f)* quality of life as assessed by paediatric health outcome and visual function questionnaires.

225 **Trial duration**

226 All patients will be assessed at baseline, 3, 6, 9, 12, 15 and 18 months. Any patient found to have
227 >1.5D increase in K_2 will need to have this confirmed at a subsequent visit (i.e. 3 months later).
228 Participants who have unconfirmed progression at the 18 month follow-up visit will need this
229 confirmed at a further visit at 21 months.

230 **Adverse events**

231 Patients will be assessed for adverse events at the one week post-CXL follow-up and at all visits
232 following randomisation.

233 *(i)* Any reversible or short-term corneal abnormality, e.g. prolonged eye pain, delayed corneal
234 epithelialisation, transient corneal oedema.

235 *(ii)* Any visually significant corneal abnormality, e.g. opacity resulting from sterile inflammatory
236 infiltrates, corneal infection or stromal melting.

237 *(iii)* Any untoward medical occurrence in a study patient which does not necessarily have a causal
238 relationship with the treatment under study, e.g. abnormal laboratory findings, or disease symptoms
239 and signs.

240 The Independent Data Monitoring Committee (IDMC) will monitor adverse events and serious
241 adverse events during the trial to inform their recommendations to the Trial Steering Committee
242 (TSC). Participants in the Standard Care arm with significant progression confirmed at two successive
243 examinations will be considered for other keratoconus management options including cross-over to
244 CXL

245 **Sample size calculation**

246 The primary outcome is K_2 at 18 months, adjusted for K_2 at baseline, in the study eye recorded by an
247 optometrist masked to the treatment group. A difference between the groups in the change in K_2 of

For peer review only - http://bmjopen.bmj.com/site/about/guidelines.xhtml

> 1.5D from randomisation to 18 months is considered to be a clinically important difference (based on Wittig-Silva et al[9]). A K_2 increase >1.5D would discriminate a true change in the steepest corneal meridian from measurement artefact and would be visually significant. A sample size of 46 patients would be required to detect this difference at the 5% significance level with 90% power, assuming a SD of 1.5D. The total sample size has been increased to 60 patients (30 per group) to allow for up to 24% loss to follow-up. These estimates are based on 12 and 24 month data reported by Wittig-Silva et al from which we estimated a pooled SD of the changes of 1.476D. We expect that on average there will be 10% loss to follow-up in both groups. In the study by Wittig-Silva et al, 19% of patients withdrew, crossed over to CXL or had a transplant by 18 months. However, 18% of patients in the control group either received CXL or a transplant. If we specifically adjust the sample size to take account of 10% loss to follow up and up to 20% of the control arm cross-over to CXL or transplant, then our planned total sample size of 60 patients would still provide at least 80% power to detect the clinically important difference. The trial protocol states that participants cannot cross over to CXL before 9 months.

Patient partnership strategy

Patients and parents were first involved in this research at a patient event hosted by Moorfields Eye Hospital. Topics on which opinions were collected included randomisation, cross-over and the duration of follow up of trial patients. The research questions and trial outcome measures in the protocol were finalised following this meeting and additional input from the UK Keratoconus Self-Help and Support Association. The Association supported the trial by providing representatives on the trial management group and the trial independent data monitoring committee. The Association will also disseminate in their website and other communications the results to participants and keratoconus patients.

Statistical analysis plan

For peer review only - http://bmjopen.bmj.com/site/about/guidelines.xhtml

The primary analysis will be conducted following the intention to treat (ITT) principle where all randomised patients will be analysed in their allocated group whether or not they receive their allocated treatment. Patient characteristics at the time of randomisation will be summarised using mean and standard deviation for continuous variables which are approximately normally distributed, median and interquartile range for variables which are not normally distributed, or by frequencies and percentages for categorical variables. All statistical tests will use a 2-sided *p*-value of 0.05 unless otherwise specified. All confidence intervals presented will be 95 % and two-sided. A detailed statistical analysis plan will be developed for approval by the TSC and review by the IDMC and finalised before the first statistical analysis of unmasked outcome measures. No formal interim analysis is planned, but reports concerning patient safety and key efficacy outcomes will be prepared for regular review by the IDMC who may request an interim analysis if a report raises concern. The IDMC is independent from the sponsor and funders. The membership, frequency of meetings, activity (including trial conduct and data review) and authority will be covered in the UCL CCTU IDMC terms of reference.

For each patient the eye with the more advanced keratoconus at the time of randomisation will be defined as the study eye for the primary analysis, unless that eye has previously been treated by CXL or corneal transplantation. The analysis of the primary outcome will be performed using a linear mixed model fitted to all K_2 values recorded after randomisation. K_2 at randomisation, treatment group, follow-up time, the interaction between treatment and time, and the stratifying variables centre and whether each patient has one or both eyes eligible will be included as fixed effects. A random patient effect will be included to take account of clustering by patient. The regression coefficient for treatment group in this model estimates the difference between the mean changes in K_2 of each group[16]. Model assumptions will be assessed, and a logarithmic transformation may be used if this improves normality of the residuals. In the event of substantial (>10%) cross-over from the randomised arm to the other arm, we will perform two analyses of the primary outcome, the primary ITT analysis and a per protocol analysis. The per-protocol analysis will exclude any

For peer review only - http://bmjopen.bmj.com/site/about/guidelines.xhtml

information collected from a patient after cross-over. Any cross-over or other treatment deviations will be summarised with reasons.

An ITT analysis will be performed for all secondary outcomes. Secondary continuous outcomes such as uncorrected and best corrected visual acuity measured at randomisation and on more than one occasion during follow-up will be analysed using similar linear mixed models. Uncorrected and best corrected visual acuity will be measured in logMAR using an ETDRS chart at a distance of 4 metres. In patients for whom both eyes show progression at the time of randomisation, information from both eyes will be included in a secondary analysis including eye as a fixed effect and patient as a random effect.

Fisher's exact test will be used to compare the proportion of study eyes with keratoconus progression in each treatment group. Cox regression analysis will be used to estimate time to keratoconus progression in the study eye for each treatment group. The model will adjust for the stratifying variables, centre and whether each patient has one or both eyes eligible. Patients who do not progress during the course of the trial will be censored at their last follow-up visit.

We will also explore how visual disability and health in children and young patients with keratoconus relate to changes in K_2. The impact of missing data will be mitigated against by incorporating information from all observed time points using a mixed model approach.

Planned subgroup analyses will be conducted to investigate whether the effect of CXL differs between patients who had progression at randomisation in one or both eyes. This will be explored by adding an interaction between the number of eyes with progression at randomisation and CXL treatment to the primary efficacy outcome analysis mixed model.

ETHICS AND DISSEMINATION

Ethical and safety considerations

The trial was approved by the UK Health Research Authority and the Medicines and Healthcare Regulatory Agency. Ethics approval was granted by the Brent Ethics Committee (reference

For peer review only

For peer review only - http://bmjopen.bmj.com/site/about/guidelines.xhtml

16/LO/0913). Trial investigators will ensure that the study (including any approved amendments) is conducted in accordance with the principles of the Declaration of Helsinki.

Dissemination plan

The results of the trial will be reported in accordance with CONSORT guidance and will be disseminated regardless of the direction of effect. Publications generated from the trial will be attributed to the trial management group (TMG), which consists of all those who have wholeheartedly collaborated in the trial. The main report will be drafted by the TMG, and the final version will be reviewed by the trial steering committee before submission for publication. Trial findings will be disseminated to the patients, UK Keratoconus Self-Help and Support Group and also doctors, optometrists, advisory bodies and healthcare commissioners. This will take the form of papers in peer-reviewed open-access medical journals and presentations at conferences.

For peer review only

REFERENCES

1. Nielsen K, Hjortdal J, Aagaard Nohr E, Ehlers N. Incidence and prevalence of keratoconus in Denmark. *Acta Ophthalmol Scand* 2007;85:890-2.
2. Godefrooij DA, Ardine de Wit G, Uiterwaal CS, Imhof SA, Wisse RPL. Age-specific incidence and prevalence of keratoconus: A nationwide registration study. *Am J Ophthalmol* 2017;175:169-172.
3. Tuft SJ, Moodaley LC, Gregory WM, et al. Prognostic factors for the progression of keratoconus. *Ophthalmology* 1994;101:439-447.
4. Léoni-Mesplié S, Mortemousque B, Touboul D, Malet F, Praud D, Mesplié N, Colin J. Scalability and severity of keratoconus in children. *Am J Ophthalmol* 2012;154:56-62.
5. Wollensak G, Spoerl E, Seiler T. Riboflavin/ultraviolet-A induced collagen crosslinking for the treatment of keratoconus. *Am J Ophthalmol* 2003; 135(5): 620–627.
6. Henriquez M A, Izquierdo Jr L, Bernilla C, Zakrzewski PA, Mannis M. Riboflavin/Ultraviolet A corneal collagen cross-linking for the treatment of keratoconus: visual outcomes and Scheimpflug analysis. *Cornea* 2011;30:281–6.

For peer review only - http://bmjopen.bmj.com/site/about/guidelines.xhtml

7. Hersh PS, Greenstein SA, Fry K. Corneal collagen cross-linking for keratoconus and corneal ectasia: One-year results. *J Cat Refract Surg* 2011;37:149-60.

8. O'Brart DP, Chan E, Samaras K, Patel P, Shah SP. A randomised, prospective study to investigate the efficacy of riboflavin/ultraviolet A (370 nm) corneal collagen cross-linkage to halt the progression of keratoconus. *Br J Ophthalmol* 2011;95:1519-24.

9. Wittig-Silva C, Chan E, Islam FM, Wu T, Whiting M, Snibson GR. A randomized, controlled trial of corneal collagen cross-linking in progressive keratoconus: three-year results. *Ophthalmology* 2014;121:812-21.

10. Sykakis E, Karim R, Evans JR, Bunce C, Amissah-Arthur KN, Patwary S, McDonnell PJ, Hamada S. Corneal collagen cross-linking for treating keratoconus. *Cochrane Database of Systematic Reviews* 2015, Issue 3. Art. no.: CD010621. DOI: 10.1002/14651858.CD010621.pub2.

11. Mazzotta C, Traversi C, Baiocchi S, Bagaglia S, Caporossi O, Villano A, Caporossi A. Corneal collagen cross-linking with riboflavin and ultraviolet A light for pediatric keratoconus: ten year results. *Cornea* 2018;37;560.

12. Vinciguerra P, Albé E, Frueh B, Trazza S, Epstein D. Two-year corneal cross-linking results in patients younger than 18 years with documented progressive keratoconus. *Am J Ophthalmol* 2012;154:520–6.

13. Godefrooij DA, Soeters N, Imhof SM, Wisse RPL. Corneal cross-linking for pediatric keratoconus: long-term results. *Cornea* 2016;35:954-958.

14. Khadka J, Ryan B, Margrain TH, Court H, Woodhouse JM. Development of the 25-item Cardiff Visual Ability Questionnaire for Children (CVAQC). *Br J Ophthalmol* 2010;94:730-5.

15. Ratcliffe J, Stevens K, Flynn T, Brazier J, Sawyer M. An assessment of the construct validity of the CHU9D in the Australian adolescent general population. *Quality of Life Res* 2012;21:717-25.

16. Vickers AJ, Altman DG. Analysing controlled trials with baseline and follow up measurements. *Br Med J* 2001;323:1123-4.

For peer review only - http://bmjopen.bmj.com/site/about/guidelines.xhtml

AUTHORS' CONTRIBUTIONS

DFPL was responsible for the trial concept. All authors made substantial contributions to the design of the study and protocol. DFPL drafted the manuscript based on the KERALINK trial protocol. KC and CD drafted the statistical analysis methods, and all authors provided critical review and approved the final manuscript. Consent for publication is given by all authors.

FUNDING STATEMENT

This work was supported by the Efficacy and Mechanism Evaluation Programme (reference 14/23/18), a MRC and NIHR partnership. The trial sponsor is University College London (contact Emilia.caverly@ucl.ac.uk). The funding organisation had no role in the design or conduct of this research. This report presents independent research commissioned by the NIHR; the views and opinions expressed by authors in this publication are those of the authors and do not necessarily reflect those of the MRC, NIHR, the EME programme or the Department of Health. This research was otherwise supported in part by the NIHR Moorfields Biomedical Research Centre and the NIHR Moorfields Clinical Research Facility, London, United Kingdom.

COMPETING INTERESTS

None.

For peer review only

FLOW DIAGRAM

KERALINK: EFFICACY AND SAFETY OF CROSS-LINKING IN CHILDREN WITH KERATOCONUS

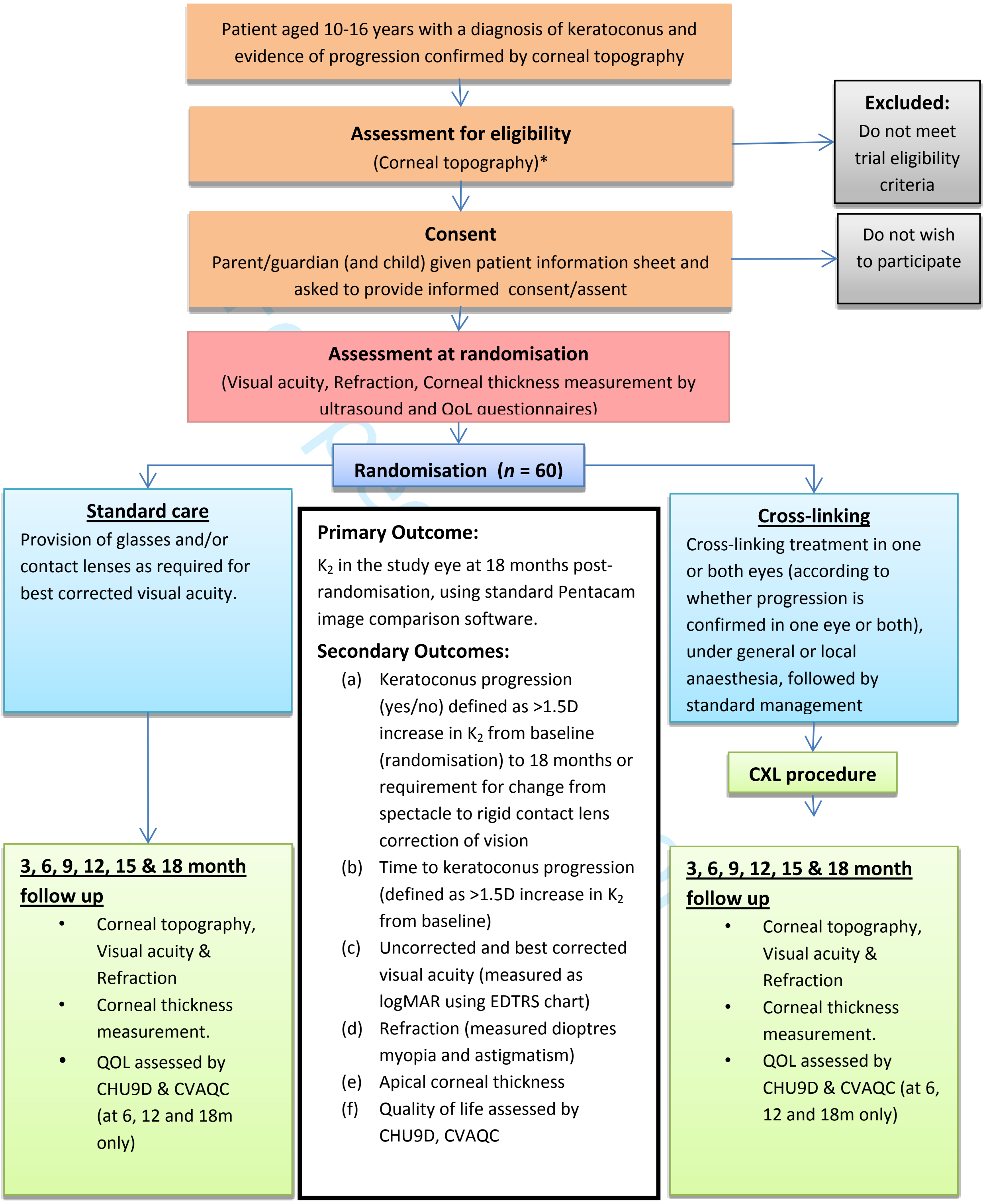

Reporting checklist for protocol of a clinical trial.

Based on the SPIRIT guidelines.

Instructions to authors

Complete this checklist by entering the page numbers from your manuscript where readers will find each of the items listed below.

Your article may not currently address all the items on the checklist. Please modify your text to include the missing information. If you are certain that an item does not apply, please write "n/a" and provide a short explanation.

Upload your completed checklist as an extra file when you submit to a journal.

In your methods section, say that you used the SPIRIT reporting guidelines, and cite them as:

Chan A-W, Tetzlaff JM, Altman DG, Laupacis A, Gøtzsche PC, Krleža-Jerić K, Hróbjartsson A, Mann H, Dickersin K, Berlin J, Doré C, Parulekar W, Summerskill W, Groves T, Schulz K, Sox H, Rockhold FW, Rennie D, Moher D. SPIRIT 2013 Statement: Defining standard protocol items for clinical trials. Ann Intern Med. 2013;158(3):200-207

For peer review only

		Reporting Item	Page Number
Title	#1	Descriptive title identifying the study design, population, interventions, and, if applicable, trial acronym	1
Trial registration	#2a	Trial identifier and registry name. If not yet registered, name of intended registry	3

Trial registration: data set	#2b	All items from the World Health Organization Trial Registration Data Set	7
Protocol version	#3	Date and version identifier	3
Funding	#4	Sources and types of financial, material, and other support	16
Roles and responsibilities: contributorship	#5a	Names, affiliations, and roles of protocol contributors	16
Roles and responsibilities: sponsor contact information	#5b	Name and contact information for the trial sponsor	16
Roles and responsibilities: sponsor and funder	#5c	Role of study sponsor and funders, if any, in study design; collection, management, analysis, and interpretation of data; writing of the report; and the decision to submit the report for publication, including whether they will have ultimate authority over any of these activities	16
Roles and responsibilities: committees	#5d	Composition, roles, and responsibilities of the coordinating centre, steering committee, endpoint adjudication committee, data management team, and other individuals or groups overseeing the trial, if applicable (see Item 21a for data monitoring committee)	11,13
Background and rationale	#6a	Description of research question and justification for undertaking the trial, including summary of relevant studies	7

For peer review only

		(published and unpublished) examining benefits and harms for each intervention	
Background and rationale: choice of comparators	#6b	Explanation for choice of comparators	5,6
Objectives	#7	Specific objectives or hypotheses	6
Trial design	#8	Description of trial design including type of trial (eg, parallel group, crossover, factorial, single group), allocation ratio, and framework (eg, superiority, equivalence, non-inferiority, exploratory)	7-8
Study setting	#9	Description of study settings (eg, community clinic, academic hospital) and list of countries where data will be collected. Reference to where list of study sites can be obtained	7
Eligibility criteria	#10	Inclusion and exclusion criteria for participants. If applicable, eligibility criteria for study centres and individuals who will perform the interventions (eg, surgeons, psychotherapists)	8
Interventions: description	#11a	Interventions for each group with sufficient detail to allow replication, including how and when they will be administered	9-10
Interventions: modifications	#11b	Criteria for discontinuing or modifying allocated interventions for a given trial participant (eg, drug dose change in response to harms, participant request, or	11

For peer review only

		improving / worsening disease)	
Interventions: adherance	#11c	Strategies to improve adherence to intervention protocols, and any procedures for monitoring adherence (eg, drug tablet return; laboratory tests)	n/a
Interventions: concomitant care	#11d	Relevant concomitant care and interventions that are permitted or prohibited during the trial	10
Outcomes	#12	Primary, secondary, and other outcomes, including the specific measurement variable (eg, systolic blood pressure), analysis metric (eg, change from baseline, final value, time to event), method of aggregation (eg, median, proportion), and time point for each outcome. Explanation of the clinical relevance of chosen efficacy and harm outcomes is strongly recommended	10-11
Participant timeline	#13	Time schedule of enrolment, interventions (including any run-ins and washouts), assessments, and visits for participants. A schematic diagram is highly recommended (see Figure)	Figure 1
Sample size	#14	Estimated number of participants needed to achieve study objectives and how it was determined, including clinical and statistical assumptions supporting any sample size calculations	12
Recruitment	#15	Strategies for achieving adequate participant enrolment to reach target sample size	n/a

For peer review only

Allocation: sequence generation	#16a	Method of generating the allocation sequence (eg, computer-generated random numbers), and list of any factors for stratification. To reduce predictability of a random sequence, details of any planned restriction (eg, blocking) should be provided in a separate document that is unavailable to those who enrol participants or assign interventions	9
Allocation concealment mechanism	#16b	Mechanism of implementing the allocation sequence (eg, central telephone; sequentially numbered, opaque, sealed envelopes), describing any steps to conceal the sequence until interventions are assigned	9
Allocation: implementation	#16c	Who will generate the allocation sequence, who will enrol participants, and who will assign participants to interventions	9
Blinding (masking)	#17a	Who will be blinded after assignment to interventions (eg, trial participants, care providers, outcome assessors, data analysts), and how	8
Blinding (masking): emergency unblinding	#17b	If blinded, circumstances under which unblinding is permissible, and procedure for revealing a participant's allocated intervention during the trial	n/a
Data collection plan	#18a	Plans for assessment and collection of outcome, baseline, and other trial data, including any related processes to promote data quality (eg, duplicate measurements, training of assessors) and a description of study instruments (eg,	9

For peer review only

		questionnaires, laboratory tests) along with their reliability and validity, if known. Reference to where data collection forms can be found, if not in the protocol	
Data collection plan: retention	#18b	Plans to promote participant retention and complete follow-up, including list of any outcome data to be collected for participants who discontinue or deviate from intervention protocols	n/a
Data management	#19	Plans for data entry, coding, security, and storage, including any related processes to promote data quality (eg, double data entry; range checks for data values). Reference to where details of data management procedures can be found, if not in the protocol	9
Statistics: outcomes	#20a	Statistical methods for analysing primary and secondary outcomes. Reference to where other details of the statistical analysis plan can be found, if not in the protocol	12-14
Statistics: additional analyses	#20b	Methods for any additional analyses (eg, subgroup and adjusted analyses)	14
Statistics: analysis population and missing data	#20c	Definition of analysis population relating to protocol non-adherence (eg, as randomised analysis), and any statistical methods to handle missing data (eg, multiple imputation)	13
Data monitoring: formal committee	#21a	Composition of data monitoring committee (DMC); summary of its role and reporting structure; statement of whether it is independent from the sponsor and competing interests; and reference to where further details about its charter can be	13

For peer review only

		found, if not in the protocol. Alternatively, an explanation of why a DMC is not needed	
Data monitoring: interim analysis	#21b	Description of any interim analyses and stopping guidelines, including who will have access to these interim results and make the final decision to terminate the trial	n/a
Harms	#22	Plans for collecting, assessing, reporting, and managing solicited and spontaneously reported adverse events and other unintended effects of trial interventions or trial conduct	11
Auditing	#23	Frequency and procedures for auditing trial conduct, if any, and whether the process will be independent from investigators and the sponsor	n/a
Research ethics approval	#24	Plans for seeking research ethics committee / institutional review board (REC / IRB) approval	14-15
Protocol amendments	#25	Plans for communicating important protocol modifications (eg, changes to eligibility criteria, outcomes, analyses) to relevant parties (eg, investigators, REC / IRBs, trial participants, trial registries, journals, regulators)	15
Consent or assent	#26a	Who will obtain informed consent or assent from potential trial participants or authorised surrogates, and how (see Item 32)	9
Consent or assent: ancillary studies	#26b	Additional consent provisions for collection and use of participant data and biological specimens in ancillary studies, if applicable	n/a

For peer review only

Confidentiality	#27	How personal information about potential and enrolled participants will be collected, shared, and maintained in order to protect confidentiality before, during, and after the trial	n/a
Declaration of interests	#28	Financial and other competing interests for principal investigators for the overall trial and each study site	17
Data access	#29	Statement of who will have access to the final trial dataset, and disclosure of contractual agreements that limit such access for investigators	n/a
Ancillary and post trial care	#30	Provisions, if any, for ancillary and post-trial care, and for compensation to those who suffer harm from trial participation	n/a
Dissemination policy: trial results	#31a	Plans for investigators and sponsor to communicate trial results to participants, healthcare professionals, the public, and other relevant groups (eg, via publication, reporting in results databases, or other data sharing arrangements), including any publication restrictions	15
Dissemination policy: authorship	#31b	Authorship eligibility guidelines and any intended use of professional writers	15
Dissemination policy: reproducible research	#31c	Plans, if any, for granting public access to the full protocol, participant-level dataset, and statistical code	n/a
Informed consent	#32	Model consent form and other related documentation given	n/a

For peer review only

materials		to participants and authorised surrogates	
Biological specimens	#33	Plans for collection, laboratory evaluation, and storage of biological specimens for genetic or molecular analysis in the current trial and for future use in ancillary studies, if applicable	n/a

The SPIRIT checklist is distributed under the terms of the Creative Commons Attribution License CC-BY-ND 3.0. This checklist was completed on 10. December 2018 using http://www.goodreports.org/, a tool made by the EQUATOR Network in collaboration with Penelope.ai

For peer review only

BMJ Open

A randomised, controlled, observer-masked trial of corneal cross-linking for progressive keratoconus in children: the KERALINK protocol

Journal:	*BMJ Open*
Manuscript ID	bmjopen-2018-028761.R1
Article Type:	Protocol
Date Submitted by the Author:	09-Aug-2019

Complete List of Authors:	Chowdhury, Kashfia; University College London, Comprehensive Clinical Trials Unit<br>Dore, Caroline; University College London, Comprehensive Clinical Trials Unit<br>Burr, Jennifer; University of St Andrews<br>Bunce, Catey; kings College london, Primary Care and Public Health Sciences<br>Raynor, Mathew; Royal Hallamshire Hospital, Ophthalmology<br>Edwards, Matthew; Royal Hallamshire Hospital, Ophthalmology<br>Larkin, Daniel; Moorfields Eye Hospital,

**Primary Subject Heading**:	Ophthalmology
Secondary Subject Heading:	Paediatrics
Keywords:	Corneal and external diseases < OPHTHALMOLOGY, Paediatric ophthalmology < OPHTHALMOLOGY, cross linking, keratoconus

SCHOLARONE™ Manuscripts

A randomised, controlled, observer-masked trial of corneal cross-linking for progressive keratoconus in children: the KERALINK protocol

Chowdhury K[1], Doré C[1], Burr JM[2], Bunce C[3], Raynor M[4], Edwards M[4], Larkin DFP[5] for the Keralink Trial Study Group.

Author affiliations and email addresses

[1] UCL Comprehensive Clinical Trials Unit, London

[2] University of St Andrews, Fife

[3] School of Population Health and Environmental Sciences, King's College London

[4] Royal Hallamshire Hospital, Sheffield

[5] NIHR Moorfields Biomedical Research Centre and NIHR Moorfields Clinical Research Facility, Moorfields Eye Hospital, London, United Kingdom

k.chowdhury@ucl.ac.uk

caroline.dore@ucl.ac.uk

jmb28@st-andrews.ac.uk

catey.bunce@kcl.ac.uk

Mathew.raynor@sth.nhs.uk

Matthew.edwards@sth.nhs.uk

f.larkin@ucl.ac.uk

Corresponding author

Daniel F P Larkin MD FRCPI FRCOphth
Moorfields Eye Hospital, City Road, London EC1V 2PD, UK

f.larkin@ucl.ac.uk

For peer review only - http://bmjopen.bmj.com/site/about/guidelines.xhtml

Tel 020 7566 2045

Keywords

Cornea, keratoconus, progression, cross linking, topography

Word count 3837 excluding title page and references

For peer review only

For peer review only - http://bmjopen.bmj.com/site/about/guidelines.xhtml

ABSTRACT

Introduction The Keralink trial tests the hypothesis that corneal cross-linking treatment (CXL) reduces the progression of keratoconus in comparison to standard care in patients under 17 years old. Keralink is a randomised controlled, observer-masked, multicentre trial in progressive keratoconus comparing epithelium-off CXL with standard care, including spectacles or contact lenses as necessary for best corrected acuity.

Methods and analysis A total of 30 participants will be randomised per group. Eligible participants aged 10-16 years with progressive keratoconus in one or both eyes will be recruited. Following randomisation, participants will be followed up 3-monthly for 18 months. The effect on progression will be determined by K_2 on corneal topography. The primary outcome measure is between-group difference in K_2 at 18 months adjusted for K_2 at baseline examination. Secondary outcomes are the effect of CXL on *(i)* keratoconus progression, *(ii)* time to keratoconus progression, *(iii)* visual acuity, *(iv)* refraction *(v)* apical corneal thickness and *(vi)* adverse events. Patient-reported effects will be explored by questionnaires.

Ethics and dissemination Research Ethics Committee Approval was obtained on 30 June 2016 (ref: 14/LO/1937). Current protocol: v5.0 (08/11/2017). Study findings will be published in peer-reviewed journals.

Trial registration number EudraCT 2016-001460-11 .

For peer review only

STRENGTHS AND LIMITATIONS OF THIS STUDY

- This is the first randomised trial of corneal cross-linking (CXL) in keratoconus in children, in which group disease onset is at an early age, is perceived to be at high risk of progression to corneal transplantation and in which only observational studies have been published.
- A total of 60 patients aged 10-16 years with progressive keratoconus will be randomised to CXL or standard care including spectacles and contact lenses as required for best corrected vision.
- The trial is designed to examine safety and efficacy of CXL in reducing progression, the primary outcome measure being between-group difference in K_2 at 18 months adjusted for K_2 at baseline examination and measured by masked optometrists.
- Secondary outcome measures at 18 months include keratoconus progression, visual acuity, refraction, adverse events and quality of life measurements.
- Follow up to 18 months after randomisation is relatively short and any benefit found following CXL would require longer term analysis of efficacy.

For peer review only

INTRODUCTION

Keratoconus is characterised by thinning and distortion of the cornea that results in visual loss from complex refractive error and corneal opacification. The prevalence in Europe has been reported as 1:1163[1] and 1:375[2]. The age at initial referral to hospital clinics is the second and third decade (mean age at diagnosis 28 years[2]), with progression until the early 30s in most affected eyes. In its early stages keratoconus causes worsening of vision on account of increasing myopia and irregular astigmatism: spectacle correction provides good visual acuity in early disease only, until increasing irregular astigmatism requires correction with rigid contact lenses for best vision. Patients with more advanced keratoconus lose contact lens-corrected visual acuity on account of corneal opacification and corneal transplant surgery is eventually required in more than 20% of patients[3]. Keratoconus is often more advanced when first diagnosed in children than in adults, with faster subsequent disease progression[4].

The most important parameters used in the assessment of keratoconus are the curvature of the cornea (presented as dioptre power (D)), apical corneal thickness in µm, refraction, and best-corrected visual acuity. Earliest disease can be detected by corneal topography, which demonstrates thinning and irregularity of corneal curvature. Quantification of steepness of the corneal curvature in horizontal, vertical and multiple oblique meridians identifies the meridian of maximum corneal steepness (K_2) and the point of maximum steepness (K_{max}).

While the standard care described above involves treatment of the refractive consequences of keratoconus or replacement of the diseased cornea by a transplant, the concept of stabilising keratoconus and arresting its progression at a stage when there is still good unaided or spectacle-corrected vision is relatively recent. Corneal cross-linking (CXL) increases the stiffness of the cornea, which can arrest the progression of early keratoconus[5]. It is the only current intervention for this purpose. In the epithelium-off CXL procedure corneal epithelium is removed, riboflavin eye drops

For peer review only - http://bmjopen.bmj.com/site/about/guidelines.xhtml

administered, and the cornea exposed to ultraviolet (UV) light for 8 or more minutes. CXL has been reported to be effective in arresting keratoconus progression in the majority of treated adult eyes in a number of non-randomised studies (including Henriquez et al. 2011[6], Hersh et al. 2011[7]) and randomised controlled trials (RCTs) (O'Brart et al 2011[8], Wittig-Silva et al. 2014[9]). In the larger study by Wittig-Silva et al. a significant difference in progression of corneal power in the steepest axis (termed 'K_{max} by these authors but in later publications widely designated 'K2') between CXL and control eyes was reported: an improvement in CXL-treated eyes with flattening of K_{max} by -1.03 ± 0.19 D compared to an increase in K_{max} for control eyes of +1.75 ± 0.38 D at 36 months. Adverse effects were not uncommon but mostly transient, including corneal oedema, superficial opacification and recurrent corneal erosions. Despite increasing information in relation to the efficacy of CXL a Cochrane Review conducted in 2015 concluded that evidence for the use of CXL in the management of keratoconus is limited due to the lack of properly conducted RCTs.[10]

In younger subjects a number of observational studies of CXL in keratoconus patients <19 years have been published, each with limitations but each reporting effectiveness. Caporossi et al. reported an uncontrolled study of 152 keratoconus patients ranging in age from 10 to 18 years, of whom follow-up post-CXL was available on only 61% of patients[11]. Inclusion criteria included several parameters which are well recognised to be characterised by inter-test variability. In this treated patient group, a statistically significant reduction of K_{max} by -0.4 D was found. Vinciguerra et al. reported 40 CXL-treated eyes in patients with progressive keratoconus aged 9-18 (mean 14.2) years in a non-randomised prospective study[12]. Findings included improved visual acuity, reduced myopic spherical equivalent on refraction testing and flattening on keratometry readings compared to pre-CXL. Goodfrooij et al reported progression in 22% within five years of CXL[13]. Although the findings from these studies suggested a beneficial effect of CXL, more robust evidence is required to inform practice. Of note, no randomised trial has been undertaken in young patients. The Keralink trial has been designed to investigate efficacy and safety of the established technique of CXL in progressive

For peer review only - http://bmjopen.bmj.com/site/about/guidelines.xhtml

keratoconus in the paediatric age group, in which on account of early disease onset there is such potential for keratoconus progression. This paper describes the design of the trial, which compares progression of keratoconus in a population of children and young patients randomised to CXL or standard care, and evaluates safety of the intervention in this patient group.

Evidence of effectiveness of CXL is of particular interest in young patients and has specifically been requested by the National Institute for Health and Care Excellence in the United Kingdom. Keralink is a multicentre randomised controlled trial in this patient group evaluating epithelium-off CXL, the technique of CXL which has been demonstrated to be effective in adults. If the trial demonstrates efficacy of CXL compared to standard care, and in particular if CXL is arrests keratoconus progression, this would have important implications for clinical management. Although we intend to follow up the trial patients for several years after the proposed trial concludes in order to ascertain the duration of keratoconus stability, it is clear that arrested progression in a paediatric patient is likely *(a)* to obviate the need for contact lens correction and for later corneal transplant surgery and *(b)* to have correspondingly greater health and cost benefit than if the CXL were undertaken in adults. Trial findings will inform ophthalmologists, optometrists and inform future research and treatment policy.

METHODS AND ANALYSIS

Study design

Keralink is a randomised controlled, observer-masked controlled trial in five centres in the United Kingdom. The study adheres to the tenets of the Declaration of Helsinki and is registered at www.controlled-trials.com (trial registration number: EudraCT 2016-001460-11). It was approved by the UK Health Research Authority, the Medicines and Healthcare Regulatory Agency and ethical approval was granted by the Brent Ethics Committee (reference 16/LO/0913). The trial is supervised by a trial management group, with independent oversight by a trial steering committee and a data monitoring committee. Eligible patients are randomised in a 1:1 ratio to receive either CXL or

For peer review only - http://bmjopen.bmj.com/site/about/guidelines.xhtml

For peer review only

standard care including spectacles or contact lenses as necessary (standard care of early keratoconus in the United Kingdom includes correction of refractive error and not CXL). Following randomisation, participants are followed for 18 months at 3-monthly intervals. Inclusion and exclusion criteria are shown in Table 1. All follow up measurements are performed by masked observers (optometrists) and the treating ophthalmologists are masked as to keratometry values on topography at follow up. Randomisation commenced on October 31 2016 and follow up of the last recruited patient is estimated to complete in mid-2020.

TABLE 1

Keralink inclusion and exclusion criteria

INCLUSION CRITERIA	Age at randomisation: 10-16 years
	Confirmed keratoconus diagnosis
	Progression on Pentacam topography in one or both eyes, steepest corneal meridian (K2) or K_{max} >1.5D
EXCLUSION CRITERIA	Apical scarring
	Cone apex thickness <400µm
	K2 >62.0 D or K_{max} >70.0 D
	Rigid lens wear in both eyes and unable to abstain for 7 days pre-topography examinations
	Down's syndrome

Definition of progression for eligibility

To differentiate true keratoconus progression from measurement artefact or minimal progression, an increase on topography (Pentacam, Oculus GmbH, Wetzlar, Germany) in the steepest keratometry (K_{max}) or in the steepest corneal meridian (K_2) of at least 1.5 dioptres (D) was used as threshold for eligibility in one or both eyes. Based on this, eligibility was defined by an increase from baseline in K_{max} or K_2 of >1.5D between two topography examinations separated by 3 or more months. For each patient the eye with the more advanced keratoconus at baseline will be categorised as the study eye for the primary analysis, unless that eye had undergone prior surgery such as corneal transplantation.

For peer review only

For peer review only - http://bmjopen.bmj.com/site/about/guidelines.xhtml

Baseline assessment

At baseline all patients will be assessed as follows.

On these visits the following assessments will be performed.

- Corneal topography for measurement of corneal power in the steepest meridian (K_2), used for assessment of the primary outcome. To improve repeatability, three measurements of each eye will be taken at baseline and follow-up examinations and the mean used in comparisons. Contact lenses will be removed at least 7 days prior to topography.
- Visual acuity (unaided, spectacle- and contact lens-corrected as applicable), logMAR measured using the ETDRS chart at a starting distance of 4m in both eyes
- Subjective refraction, both eyes
- Apical corneal thickness measurement, both eyes, by ultrasound and Scheimpflug imaging at topography
- Quality of life will be assessed by visual function (CVAQC) and generic paediatric health outcome (CHU9D) questionnaires. CVAQC is a 25-item vision specific questionnaire designed for children[14]. CHU9D is a nine-question paediatric generic preference based measure of health outcome which provides a descriptive health profile as well as a utility score and has been validated for self-completion in an adolescent population (11-17 years)[15].

Randomisation and allocation of participants to treatment groups

Randomisation will be by a centralised computer generated randomisation service (*https://www.sealedenvelope.com*). The system is customised to trial requirements, using minimisation with stratification by treatment centre and whether progression is confirmed in one eye or both eyes at randomisation. Following a dedicated consent/screening and randomisation visit for eligible patients and their parents, patients will be randomised to one of two trial arms (Figure 1). Specific study information sheets will be provided to parents and patients prior to taking consent; a parent or guardian will be asked to provide consent in all cases and 15-16 year old patients will be

For peer review only - http://bmjopen.bmj.com/site/about/guidelines.xhtml

asked to provide assent if this is their choice.

Intervention: CXL

Corneal cross-linking in one or both eyes (according to whether progression is confirmed in one eye or both), under general or local anaesthesia as applicable, followed by standard management. The surgical procedure will be as follows: insertion of lid speculum, removal of corneal epithelium with a spatula, administration of riboflavin drops (Vibex Rapid, Avedro Inc., Waltham, Massachusetts, USA) every 2 minutes for 10 minutes, application of pulsed ultraviolet light using standardised parameters of 10mW/cm^2 for a 5.4J/cm^2 total energy dose administered over 8 minutes in a pulsed manner (Avedro KXL). At completion of the procedure one drop of povidone iodine and a therapeutic contact lens will be applied to the treated eye. Management post-CXL is *(i)* proxymetacaine drops every 2 hours and naproxen 250mg twice daily, both as required for analgesia, *(ii)* moxifloxacin 0.5% drops every 6 hours for one week as infection prophylaxis, *(iii)* dexamethasone 0.1% drops every 6 hours for one week, every 12 hours for one week, then fluorometholone 0.1% drops every 12 hours for one week. Patients randomised to CXL will attend for an extra examination at 1 week post-CXL for removal of the contact lens and confirmation of corneal re-epithelialisation.

Comparator: Standard care

The trial control arm is standard management alone, including refraction testing with provision of glasses and/or contact lens fitting for one or both eyes as required for best corrected visual acuity.

Defining keratoconus progression for secondary outcomes

To differentiate true keratoconus progression from measurement artefact, we will define progression as an increase in power in the steepest corneal meridian (K_2) of >1.5 D on corneal topography between two examinations or the requirement for change from spectacle to rigid contact lenses correction of vision, as the latter precludes reliable topography measurements.

Outcome measures

The primary trial outcome measure will be between-group difference in K_2 at 18 months adjusted for K_2 at baseline examination.

For peer review only - http://bmjopen.bmj.com/site/about/guidelines.xhtml

Secondary outcomes will be the effect of CXL on

(a) Keratoconus progression (yes/no) defined as >1.5D increase from baseline in K_2, confirmed at subsequent visits *or* keratoconus progression requiring change from spectacle to rigid contact lens correction of vision, which precludes reliable topography measurements

(b) time to keratoconus progression

(c) uncorrected and best corrected visual acuity (logMAR) measured with an ETDRS chart at a starting distance of 4m

(d) refraction (measured dioptres spherical equivalent, myopia and astigmatism)

(e) apical corneal thickness

(f) quality of life as assessed by paediatric health outcome and visual function questionnaires.

Trial duration

All patients will be assessed at baseline, 3, 6, 9, 12, 15 and 18 months. Any patient found to have >1.5D increase in K_2 will need to have this confirmed at a subsequent visit (i.e. 3 months later). Participants who have unconfirmed progression at the 18 month follow-up visit will need this confirmed at a further visit at 21 months.

Adverse events

Patients will be assessed for adverse events at the one week post-CXL follow-up and at all visits following randomisation.

(i) Any reversible or short-term corneal abnormality, e.g. prolonged eye pain, delayed corneal epithelialisation, transient corneal oedema.

(ii) Any visually significant corneal abnormality, e.g. opacity resulting from sterile inflammatory infiltrates, corneal infection or stromal melting.

(iii) Any untoward medical occurrence in a study patient which does not necessarily have a causal relationship with the treatment under study, e.g. abnormal laboratory findings, or disease symptoms and signs.

The Independent Data Monitoring Committee (IDMC) will monitor adverse events and serious

For peer review only - http://bmjopen.bmj.com/site/about/guidelines.xhtml

adverse events during the trial to inform their recommendations to the Trial Steering Committee (TSC). Participants in the Standard Care arm with significant progression confirmed at two successive examinations will be considered for other keratoconus management options including cross-over to CXL

Sample size calculation

The primary outcome is K_2 at 18 months, adjusted for K_2 at baseline, in the study eye recorded by an optometrist masked to the treatment group. A difference between the groups in the change in K_2 of > 1.5D from randomisation to 18 months is considered to be a clinically important difference (based on Wittig-Silva et al[9]). A K_2 increase >1.5D would discriminate a true change in the steepest corneal meridian from measurement artefact and would be visually significant. A sample size of 46 patients would be required to detect this difference at the 5% significance level with 90% power, assuming a SD of 1.5D. The total sample size has been increased to 60 patients (30 per group) to allow for up to 24% loss to follow-up. These estimates are based on 12 and 24 month data reported by Wittig-Silva et al from which we estimated a pooled SD of the changes of 1.476D. We expect that on average there will be 10% loss to follow-up in both groups. In the study by Wittig-Silva et al, 19% of patients withdrew, crossed over to CXL or had a transplant by 18 months. However, 18% of patients in the control group either received CXL or a transplant. If we specifically adjust the sample size to take account of 10% loss to follow up and up to 20% of the control arm cross-over to CXL or transplant, then our planned total sample size of 60 patients would still provide at least 80% power to detect the clinically important difference. The trial protocol states that participants cannot cross over to CXL before 9 months.

Patient and Public Involvement

Patients and parents were first involved in this research at a patient event hosted by Moorfields Eye Hospital. Topics on which opinions were collected included randomisation, cross-over and the duration of follow up of trial patients. The research questions, design and trial outcome measures in the protocol were finalised following the above meeting and additional input from the UK Keratoconus Self-Help and Support Association. This Association supported the trial by publicising

For peer review only - http://bmjopen.bmj.com/site/about/guidelines.xhtml

273 the trial and by providing representatives on the trial management group and the trial independent data monitoring committee. The investigators will communicate a summary of the trial results to participants and their parents. The UK Keratoconus Self Help and Support Association will disseminate in their website and other communications the results to keratoconus patients. The burden of the intervention was discussed at our initial meeting with patients and parents and at the consent-taking stage in the trial.

Statistical analysis plan

The primary analysis will be conducted following the intention to treat (ITT) principle where all randomised patients will be analysed in their allocated group whether or not they receive their allocated treatment. Patient characteristics at the time of randomisation will be summarised using mean and standard deviation for continuous variables which are approximately normally distributed, median and interquartile range for variables which are not normally distributed, or by frequencies and percentages for categorical variables. All statistical tests will use a 2-sided *p*-value of 0.05 unless otherwise specified. All confidence intervals presented will be 95 % and two-sided. A detailed statistical analysis plan will be developed for approval by the TSC and review by the IDMC and finalised before the first statistical analysis of unmasked outcome measures. No formal interim analysis is planned, but reports concerning patient safety and key efficacy outcomes will be prepared for regular review by the IDMC who may request an interim analysis if a report raises concern. The IDMC is independent from the sponsor and funders. The membership, frequency of meetings, activity (including trial conduct and data review) and authority will be covered in the UCL CCTU IDMC terms of reference.

For each patient the eye with the more advanced keratoconus at the time of randomisation will be defined as the study eye for the primary analysis, unless that eye has previously been treated by CXL or corneal transplantation. The analysis of the primary outcome will be performed using a linear mixed model fitted to all K_2 values recorded after randomisation. K_2 at randomisation, treatment group, follow-up time, the interaction between treatment and time, and the stratifying variables

For peer review only - http://bmjopen.bmj.com/site/about/guidelines.xhtml

centre and whether each patient has one or both eyes eligible will be included as fixed effects. A random patient effect will be included to take account of clustering by patient. The regression coefficient for treatment group in this model estimates the difference between the mean changes in K_2 of each group[16]. Model assumptions will be assessed, and a logarithmic transformation may be used if this improves normality of the residuals. In the event of substantial (>10%) cross-over from the randomised arm to the other arm, we will perform two analyses of the primary outcome, the primary ITT analysis and a per protocol analysis. The per-protocol analysis will exclude any information collected from a patient after cross-over. Any cross-over or other treatment deviations will be summarised with reasons.

An ITT analysis will be performed for all secondary outcomes. Secondary continuous outcomes such as uncorrected and best corrected visual acuity measured at randomisation and on more than one occasion during follow-up will be analysed using similar linear mixed models. Uncorrected and best corrected visual acuity will be measured in logMAR using an ETDRS chart at a distance of 4 metres. In patients for whom both eyes show progression at the time of randomisation, information from both eyes will be included in a secondary analysis including eye as a fixed effect and patient as a random effect.

Fisher's exact test will be used to compare the proportion of study eyes with keratoconus progression in each treatment group. Cox regression analysis will be used to estimate time to keratoconus progression in the study eye for each treatment group. The model will adjust for the stratifying variables, centre and whether each patient has one or both eyes eligible. Patients who do not progress during the course of the trial will be censored at their last follow-up visit.

We will also explore how visual disability and health in children and young patients with keratoconus relate to changes in K_2. The impact of missing data will be mitigated against by incorporating information from all observed time points using a mixed model approach.

Planned subgroup analyses will be conducted to investigate whether the effect of CXL differs between patients who had progression at randomisation in one or both eyes. This will be explored

For peer review only - http://bmjopen.bmj.com/site/about/guidelines.xhtml

by adding an interaction between the number of eyes with progression at randomisation and CXL treatment to the primary efficacy outcome analysis mixed model.

ETHICS AND DISSEMINATION

Ethical and safety considerations

The trial was approved by the UK Health Research Authority and the Medicines and Healthcare Regulatory Agency. Ethics approval was granted by the Brent Ethics Committee (reference 16/LO/0913). Trial investigators will ensure that the study (including any approved amendments) is conducted in accordance with the principles of the Declaration of Helsinki.

Dissemination plan

The results of the trial will be reported in accordance with CONSORT guidance and will be disseminated regardless of the direction of effect. Publications generated from the trial will be attributed to the trial management group (TMG), which consists of all those who have wholeheartedly collaborated in the trial. The main report will be drafted by the TMG, and the final version will be reviewed by the trial steering committee before submission for publication. Trial findings will be disseminated to the patients, UK Keratoconus Self-Help and Support Group and also doctors, optometrists, advisory bodies and healthcare commissioners. This will take the form of papers in peer-reviewed open-access medical journals and presentations at conferences.

KERALINK Data Availability Statement

1. Will individual participant data be available (including data dictionaries)?
Yes

2. What data in particular will be shared?
Individual participant data that underlie the results reported in this article, after de-identification and appropriate statistical disclosure control using non-perturbative methods (mainly recoding categorical variables and removing variables).

3 What other documents will be available?
Study protocol

4. When will data be available (start and end dates)?
Beginning 6 months following article publication. No end date.

For peer review only

5. With whom?

Investigators whose proposed use of the data has been approved by an internal review committee identified for this purpose.

6. For what types of analyses?

To achieve aims in the approved proposal.

7. By what mechanism will data be made available?

Proposals should be directed to cctu-enquiries@ucl.ac.uk. To gain access, a data sharing agreement will be signed. Data will be shared by an appropriate secure exchange facility.

FIGURE LEGEND

FLOW DIAGRAM

KERALINK: EFFICACY AND SAFETY OF CROSS-LINKING IN CHILDREN WITH KERATOCONUS

REFERENCES

1. Nielsen K, Hjortdal J, Aagaard Nohr E, Ehlers N. Incidence and prevalence of keratoconus in Denmark. *Acta Ophthalmol Scand* 2007;85:890-2.

2. Godefrooij DA, Ardine de Wit G, Uiterwaal CS, Imhof SA, Wisse RPL. Age-specific incidence and prevalence of keratoconus: A nationwide registration study. *Am J Ophthalmol* 2017;175:169-172.

3. Tuft SJ, Moodaley LC, Gregory WM, et al. Prognostic factors for the progression of keratoconus. *Ophthalmology* 1994;101:439-447.

4. Léoni-Mesplié S, Mortemousque B, Touboul D, Malet F, Praud D, Mesplié N, Colin J. Scalability and severity of keratoconus in children. *Am J Ophthalmol* 2012;154:56-62.

5. Wollensak G, Spoerl E, Seiler T. Riboflavin/ultraviolet-A induced collagen crosslinking for the treatment of keratoconus. *Am J Ophthalmol* 2003; 135(5): 620–627.

6. Henriquez M A, Izquierdo Jr L, Bernilla C, Zakrzewski PA, Mannis M. Riboflavin/Ultraviolet A corneal collagen cross-linking for the treatment of keratoconus: visual outcomes and Scheimpflug analysis. *Cornea* 2011;30:281–6.

7. Hersh PS, Greenstein SA, Fry K. Corneal collagen cross-linking for keratoconus and corneal ectasia: One-year results. *J Cat Refract Surg* 2011;37:149-60.

For peer review only - http://bmjopen.bmj.com/site/about/guidelines.xhtml

For peer review only

8. O'Brart DP, Chan E, Samaras K, Patel P, Shah SP. A randomised, prospective study to investigate the efficacy of riboflavin/ultraviolet A (370 nm) corneal collagen cross-linkage to halt the progression of keratoconus. *Br J Ophthalmol* 2011;95:1519-24.

9. Wittig-Silva C, Chan E, Islam FM, Wu T, Whiting M, Snibson GR. A randomized, controlled trial of corneal collagen cross-linking in progressive keratoconus: three-year results. *Ophthalmology* 2014;121:812-21.

10. Sykakis E, Karim R, Evans JR, Bunce C, Amissah-Arthur KN, Patwary S, McDonnell PJ, Hamada S. Corneal collagen cross-linking for treating keratoconus. *Cochrane Database of Systematic Reviews* 2015, Issue 3. Art. no.: CD010621. DOI: 10.1002/14651858.CD010621.pub2.

11. Mazzotta C, Traversi C, Baiocchi S, Bagaglia S, Caporossi O, Villano A, Caporossi A. Corneal collagen cross-linking with riboflavin and ultraviolet A light for pediatric keratoconus: ten year results. *Cornea* 2018;37;560.

12. Vinciguerra P, Albé E, Frueh B, Trazza S, Epstein D. Two-year corneal cross-linking results in patients younger than 18 years with documented progressive keratoconus. *Am J Ophthalmol* 2012;154:520–6.

13. Godefrooij DA, Soeters N, Imhof SM, Wisse RPL. Corneal cross-linking for pediatric keratoconus: long-term results. *Cornea* 2016;35:954-958.

14. Khadka J, Ryan B, Margrain TH, Court H, Woodhouse JM. Development of the 25-item Cardiff Visual Ability Questionnaire for Children (CVAQC). *Br J Ophthalmol* 2010;94:730-5.

15. Ratcliffe J, Stevens K, Flynn T, Brazier J, Sawyer M. An assessment of the construct validity of the CHU9D in the Australian adolescent general population. *Quality of Life Res* 2012;21:717-25.

16. Vickers AJ, Altman DG. Analysing controlled trials with baseline and follow up measurements. *Br Med J* 2001;323:1123-4.

AUTHORS' CONTRIBUTIONS

DFPL was responsible for the trial concept. JMB, CB, MR and ME made substantial contributions to the design of the study and protocol. DFPL drafted the manuscript based on the KERALINK trial

For peer review only - http://bmjopen.bmj.com/site/about/guidelines.xhtml

protocol. KC and CD drafted the statistical analysis methods, and all authors provided critical review and approved the final manuscript. Consent for publication is given by all authors.

FUNDING STATEMENT

This work was supported by the Efficacy and Mechanism Evaluation Programme (reference 14/23/18), a MRC and NIHR partnership. The trial sponsor is University College London (contact Emilia.caverly@ucl.ac.uk). The funding organisation had no role in the design or conduct of this research. This report presents independent research commissioned by the NIHR; the views and opinions expressed by authors in this publication are those of the authors and do not necessarily reflect those of the MRC, NIHR, the EME programme or the Department of Health. This research was otherwise supported in part by the NIHR Moorfields Biomedical Research Centre and the NIHR Moorfields Clinical Research Facility, London, United Kingdom.

COMPETING INTERESTS

None.

For peer review only

For peer review only - http://bmjopen.bmj.com/site/about/guidelines.xhtml

FLOW DIAGRAM

KERALINK: EFFICACY AND SAFETY OF CROSS-LINKING IN CHILDREN WITH KERATOCONUS

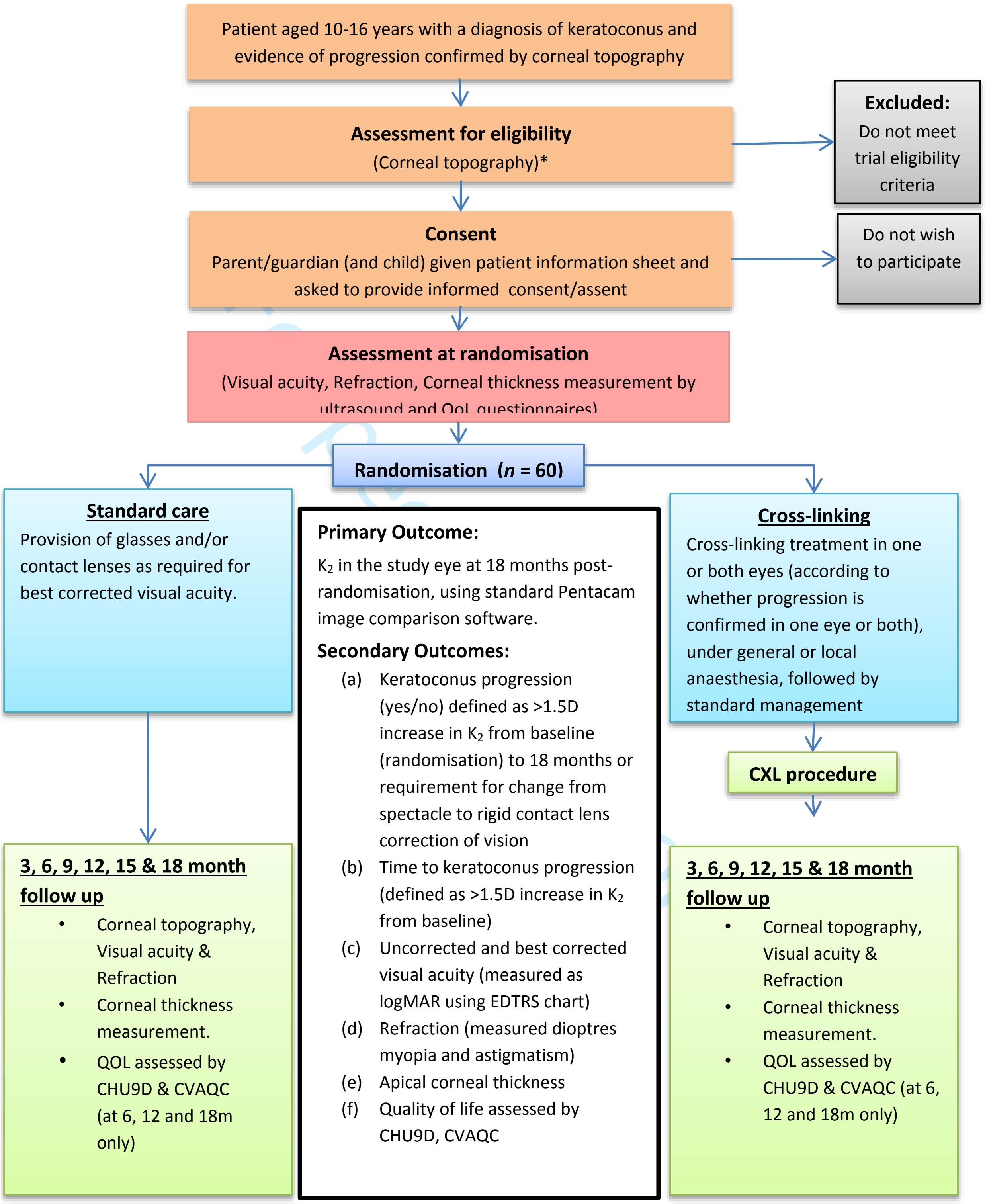

Reporting checklist for protocol of a clinical trial.

Based on the SPIRIT guidelines.

Instructions to authors

Complete this checklist by entering the page numbers from your manuscript where readers will find each of the items listed below.

Your article may not currently address all the items on the checklist. Please modify your text to include the missing information. If you are certain that an item does not apply, please write "n/a" and provide a short explanation.

Upload your completed checklist as an extra file when you submit to a journal.

In your methods section, say that you used the SPIRIT reporting guidelines, and cite them as:

Chan A-W, Tetzlaff JM, Altman DG, Laupacis A, Gøtzsche PC, Krleža-Jerić K, Hróbjartsson A, Mann H, Dickersin K, Berlin J, Doré C, Parulekar W, Summerskill W, Groves T, Schulz K, Sox H, Rockhold FW, Rennie D, Moher D. SPIRIT 2013 Statement: Defining standard protocol items for clinical trials. Ann Intern Med. 2013;158(3):200-207

For peer review only

		Reporting Item	Page Number
Title	#1	Descriptive title identifying the study design, population, interventions, and, if applicable, trial acronym	1
Trial registration	#2a	Trial identifier and registry name. If not yet registered, name of intended registry	3

Trial registration: data set	#2b	All items from the World Health Organization Trial Registration Data Set	7
Protocol version	#3	Date and version identifier	3
Funding	#4	Sources and types of financial, material, and other support	16
Roles and responsibilities: contributorship	#5a	Names, affiliations, and roles of protocol contributors	16
Roles and responsibilities: sponsor contact information	#5b	Name and contact information for the trial sponsor	16
Roles and responsibilities: sponsor and funder	#5c	Role of study sponsor and funders, if any, in study design; collection, management, analysis, and interpretation of data; writing of the report; and the decision to submit the report for publication, including whether they will have ultimate authority over any of these activities	16
Roles and responsibilities: committees	#5d	Composition, roles, and responsibilities of the coordinating centre, steering committee, endpoint adjudication committee, data management team, and other individuals or groups overseeing the trial, if applicable (see Item 21a for data monitoring committee)	11,13
Background and rationale	#6a	Description of research question and justification for undertaking the trial, including summary of relevant studies	7

For peer review only

		(published and unpublished) examining benefits and harms for each intervention	
Background and rationale: choice of comparators	#6b	Explanation for choice of comparators	5,6
Objectives	#7	Specific objectives or hypotheses	6
Trial design	#8	Description of trial design including type of trial (eg, parallel group, crossover, factorial, single group), allocation ratio, and framework (eg, superiority, equivalence, non-inferiority, exploratory)	7-8
Study setting	#9	Description of study settings (eg, community clinic, academic hospital) and list of countries where data will be collected. Reference to where list of study sites can be obtained	7
Eligibility criteria	#10	Inclusion and exclusion criteria for participants. If applicable, eligibility criteria for study centres and individuals who will perform the interventions (eg, surgeons, psychotherapists)	8
Interventions: description	#11a	Interventions for each group with sufficient detail to allow replication, including how and when they will be administered	9-10
Interventions: modifications	#11b	Criteria for discontinuing or modifying allocated interventions for a given trial participant (eg, drug dose change in response to harms, participant request, or	11

For peer review only

		improving / worsening disease)	
Interventions: adherance	#11c	Strategies to improve adherence to intervention protocols, and any procedures for monitoring adherence (eg, drug tablet return; laboratory tests)	n/a
Interventions: concomitant care	#11d	Relevant concomitant care and interventions that are permitted or prohibited during the trial	10
Outcomes	#12	Primary, secondary, and other outcomes, including the specific measurement variable (eg, systolic blood pressure), analysis metric (eg, change from baseline, final value, time to event), method of aggregation (eg, median, proportion), and time point for each outcome. Explanation of the clinical relevance of chosen efficacy and harm outcomes is strongly recommended	10-11
Participant timeline	#13	Time schedule of enrolment, interventions (including any run-ins and washouts), assessments, and visits for participants. A schematic diagram is highly recommended (see Figure)	Figure 1
Sample size	#14	Estimated number of participants needed to achieve study objectives and how it was determined, including clinical and statistical assumptions supporting any sample size calculations	12
Recruitment	#15	Strategies for achieving adequate participant enrolment to reach target sample size	n/a

For peer review only

Allocation: sequence generation	#16a	Method of generating the allocation sequence (eg, computer-generated random numbers), and list of any factors for stratification. To reduce predictability of a random sequence, details of any planned restriction (eg, blocking) should be provided in a separate document that is unavailable to those who enrol participants or assign interventions	9
Allocation concealment mechanism	#16b	Mechanism of implementing the allocation sequence (eg, central telephone; sequentially numbered, opaque, sealed envelopes), describing any steps to conceal the sequence until interventions are assigned	9
Allocation: implementation	#16c	Who will generate the allocation sequence, who will enrol participants, and who will assign participants to interventions	9
Blinding (masking)	#17a	Who will be blinded after assignment to interventions (eg, trial participants, care providers, outcome assessors, data analysts), and how	8
Blinding (masking): emergency unblinding	#17b	If blinded, circumstances under which unblinding is permissible, and procedure for revealing a participant’s allocated intervention during the trial	n/a
Data collection plan	#18a	Plans for assessment and collection of outcome, baseline, and other trial data, including any related processes to promote data quality (eg, duplicate measurements, training of assessors) and a description of study instruments (eg,	9

For peer review only

		questionnaires, laboratory tests) along with their reliability and validity, if known. Reference to where data collection forms can be found, if not in the protocol	
Data collection plan: retention	#18b	Plans to promote participant retention and complete follow-up, including list of any outcome data to be collected for participants who discontinue or deviate from intervention protocols	n/a
Data management	#19	Plans for data entry, coding, security, and storage, including any related processes to promote data quality (eg, double data entry; range checks for data values). Reference to where details of data management procedures can be found, if not in the protocol	9
Statistics: outcomes	#20a	Statistical methods for analysing primary and secondary outcomes. Reference to where other details of the statistical analysis plan can be found, if not in the protocol	12-14
Statistics: additional analyses	#20b	Methods for any additional analyses (eg, subgroup and adjusted analyses)	14
Statistics: analysis population and missing data	#20c	Definition of analysis population relating to protocol non-adherence (eg, as randomised analysis), and any statistical methods to handle missing data (eg, multiple imputation)	13
Data monitoring: formal committee	#21a	Composition of data monitoring committee (DMC); summary of its role and reporting structure; statement of whether it is independent from the sponsor and competing interests; and reference to where further details about its charter can be	13

For peer review only

		found, if not in the protocol. Alternatively, an explanation of why a DMC is not needed	
Data monitoring: interim analysis	#21b	Description of any interim analyses and stopping guidelines, including who will have access to these interim results and make the final decision to terminate the trial	n/a
Harms	#22	Plans for collecting, assessing, reporting, and managing solicited and spontaneously reported adverse events and other unintended effects of trial interventions or trial conduct	11
Auditing	#23	Frequency and procedures for auditing trial conduct, if any, and whether the process will be independent from investigators and the sponsor	n/a
Research ethics approval	#24	Plans for seeking research ethics committee / institutional review board (REC / IRB) approval	14-15
Protocol amendments	#25	Plans for communicating important protocol modifications (eg, changes to eligibility criteria, outcomes, analyses) to relevant parties (eg, investigators, REC / IRBs, trial participants, trial registries, journals, regulators)	15
Consent or assent	#26a	Who will obtain informed consent or assent from potential trial participants or authorised surrogates, and how (see Item 32)	9
Consent or assent: ancillary studies	#26b	Additional consent provisions for collection and use of participant data and biological specimens in ancillary studies, if applicable	n/a

For peer review only

Confidentiality	#27	How personal information about potential and enrolled participants will be collected, shared, and maintained in order to protect confidentiality before, during, and after the trial	n/a
Declaration of interests	#28	Financial and other competing interests for principal investigators for the overall trial and each study site	17
Data access	#29	Statement of who will have access to the final trial dataset, and disclosure of contractual agreements that limit such access for investigators	n/a
Ancillary and post trial care	#30	Provisions, if any, for ancillary and post-trial care, and for compensation to those who suffer harm from trial participation	n/a
Dissemination policy: trial results	#31a	Plans for investigators and sponsor to communicate trial results to participants, healthcare professionals, the public, and other relevant groups (eg, via publication, reporting in results databases, or other data sharing arrangements), including any publication restrictions	15
Dissemination policy: authorship	#31b	Authorship eligibility guidelines and any intended use of professional writers	15
Dissemination policy: reproducible research	#31c	Plans, if any, for granting public access to the full protocol, participant-level dataset, and statistical code	n/a
Informed consent	#32	Model consent form and other related documentation given	n/a

For peer review only

materials		to participants and authorised surrogates	
Biological specimens	#33	Plans for collection, laboratory evaluation, and storage of biological specimens for genetic or molecular analysis in the current trial and for future use in ancillary studies, if applicable	n/a

The SPIRIT checklist is distributed under the terms of the Creative Commons Attribution License CC-BY-ND 3.0. This checklist was completed on 10. December 2018 using http://www.goodreports.org/, a tool made by the EQUATOR Network in collaboration with Penelope.ai

For peer review only
